# Healthcare Use After Buprenorphine Prescription in a Community Emergency Department: A Cohort Study

**DOI:** 10.5811/westjem.2021.6.51306

**Published:** 2021-09-24

**Authors:** Tinh Le, Parker Cordial, Mackenzie Sankoe, Charlotte Purnode, Ankur Parekh, Thomas Baker, Brian Hiestand, W.F. Peacock, James Neuenschwander

**Affiliations:** * Case Western Reserve University School of Medicine, Cleveland, Ohio; † The Ohio State University College of Medicine, Columbus, Ohio; ‡ Ohio University Heritage College of Osteopathic Medicine, Athens, Ohio; § The Ohio State University, Columbus, Ohio; ¶ Genesis Healthcare System, Department of Emergency Medicine, Zanesville, Ohio; || Wake Forest School of Medicine, Department of Emergency Medicine, Winston-Salem, North Carolina; # Baylor College of Medicine, Department of Emergency Medicine, Houston, Texas

## Abstract

**Introduction:**

Recent studies from urban academic centers have shown the promise of emergency physician-initiated buprenorphine for improving outcomes in opioid use disorder (OUD) patients. We investigated whether emergency physician-initiated buprenorphine in a rural, community setting decreases subsequent healthcare utilization for OUD patients.

**Methods:**

We performed a retrospective chart review of patients presenting to a community hospital emergency department (ED) who received a prescription for buprenorphine from June 15, 2018–June 15, 2019. Demographic and opioid-related International Classification of Diseases, 10th Revision, (ICD-10) codes were documented and used to create a case-matched control cohort of demographically matched patients who presented in a similar time frame with similar ICD-10 codes but did not receive buprenorphine. We recorded 12-month rates of ED visits, all-cause hospitalizations, and opioid overdoses. Differences in event occurrences between groups were assessed with Poisson regression.

**Results:**

Overall 117 patients were included in the study: 59 who received buprenorphine vs 58 controls. The groups were well matched, both roughly 90% White and 60% male, with an average age of 33.4 years for both groups. Controls had a median two ED visits (range 0–33), median 0.5 hospitalizations (range 0–8), and 0 overdoses (range 0–3), vs median one ED visit (range 0–8), median 0 hospitalizations (range 0–4), and median 0 overdoses (range 0–3) in the treatment group. The incidence rate ratio (IRR) for counts of ED visits was 0.61, 95% confidence interval (CI), 0.49, 0.75, favoring medication-assisted treatment (MAT). For hospitalizations, IRR was 0.34, 95% CI, 0.22, 0.52 favoring MAT, and for overdoses was 1.04, 95% CI, 0.53, 2.07.

**Conclusion:**

Initiation of buprenorphine by ED providers was associated with lower 12-month ED visit and all-cause hospitalization rates with comparable overdose rates compared to controls. These findings show the ED’s potential as an initiation point for medication-assisted treatment in OUD patients.

## INTRODUCTION

The opioid epidemic is a decades-long public health crisis that is estimated to have claimed the lives of over 350,000 Americans from 1999–2016; it has far-reaching impacts beyond mortality, such as decreased quality of life, neonatal abstinence syndrome, increased healthcare utilization, and lost productivity.^1^–^4^ Unfortunately, the crisis appears to continue to accelerate, with the US Centers for Disease Control and Prevention (CDC) estimating that more Americans died of drug overdose in 2019 than in 2018, and partial data from the first half of 2019 revealing that 81.5% of recorded overdose deaths involved opioids.^5^ Even more ominously, some sources predict that the coronavirus 2019 pandemic and its consequences could worsen the opioid epidemic.^6^ This prediction is already potentially being reflected by early data.^7^

Studies have shown that medication-assisted treatment (MAT) is an effective maintenance strategy for improving quality of life, decreasing mortality, and even maintaining abstinence in some patients with opioid use disorder (OUD).^8^ These medications decrease patients’ risk of contracting infectious diseases such as human immunodeficiency virus, decrease their risk of suffering an overdose, and decrease their overall healthcare utilization.^4^, ^9^–^11^ Drugs commonly used in MAT include methadone, a full μ-opioid receptor agonist; buprenorphine, a partial μ-opioid receptor agonist; and naltrexone, a μ-opioid receptor antagonist.^12^ Due to their differing pharmacodynamics, each of these drugs has strengths and weaknesses in terms of initiation and induction, the logistics of distribution, potential for abuse, and risk of overdose and withdrawal.^12^

Buprenorphine produces mild, typical opioid effects at a low dose, but studies have shown it has a “ceiling effect,” ie, the effect does not increase as the dose is increased. In terms of safety profile, buprenorphine causes less respiratory depression than full μ agonists with lower overdose risk and less risk of arrhythmia.^13^,^14^ Buprenorphine is available in three forms: buccal or sublingual tablets; extended-release formulations (implant or depot injection); and as a skin patch, which is used for pain management.^15^ Unlike naltrexone, buprenorphine does not require a supervised withdrawal period and can be safely induced either in the emergency department (ED), the primary care setting, or at home.^16^ Unlike methadone, buprenorphine can be prescribed by any physician or advanced practice provider after undergoing proper training, and multiple days’ doses can be dispensed at once.^16^ These attributes make buprenorphine a favorable form of MAT to be prescribed by emergency physicians, attributes that become more relevant given that the ED is a key point of contact with the healthcare system for many OUD patients.^17^

Multiple recent studies have assessed the effect of buprenorphine prescription or induction by emergency physicians on patient outcomes. Most of the studies, which were conducted at urban, academic medical centers using 30-day enrollment in an MAT program as a primary endpoint, found that significant proportions of subjects attained the desired outcome.^16^,^18^–^21^ In this study we sought to determine whether buprenorphine prescription by emergency care providers in a community hospital decreased healthcare utilization in patients with OUD. We hypothesized that buprenorphine prescription by emergency care providers would safely decrease healthcare utilization for OUD patients compared to matched controls, resulting in decreased rates of ED-visit and hospitalization rates without an increase in opioid overdose rates.

**Population Health Research Capsule**

What do we already know about this issue?

*Buprenorphine initiation in the emergency department (ED) is associated with improved engagement with addiction treatment programs for patients with opioid use disorder (OUD).*

What was the research question?

*Does emergency physician-initiated buprenorphine treatment decrease healthcare utilization for OUD patients?*

What was the major finding of the study?

*Patients prescribed buprenorphine in the ED experienced significantly lower 12-month ED-visit and hospitalizations rates, but no change in overdose rate.*

How does this improve population health?

*Emergency physician-initiated buprenorphine therapy is potentially valuable both in terms of patient outcomes and overall healthcare utilization.*

## METHODS

We performed a retrospective chart-review study, which was approved by the institutional review board. The study site was a community healthcare system in the Appalachian United States with an annual ED census of 71,354. The site is the largest healthcare provider in a six-county area and is the region’s only Level III trauma center. It is also the only hospital and ED in a roughly 30-mile radius.

Emergency physicians and nurse practitioners in the hospital had undergone free 8- and 24-hour training courses, respectively, to obtain X waivers. These waivers, which can be obtained by physicians, physician assistants, nurse practitioners and other healthcare providers, allow providers to administer, dispense, and prescribe buprenorphine. Emergency care providers at the study site began prescribing buprenorphine in June 2018. As this was a retrospective analysis of buprenorphine prescription in the regular course of care, there was no formal protocol mandated for prescribing the drug to patients; providers prescribed based on their personal judgment and experience. If the choice was made to prescribe buprenorphine-naloxone or buprenorphine alone, the patient received a dose in the ED and was provided with a referral to an MAT clinic and a bridge prescription of 1–3 days.

We compiled a convenience sample of all patients prescribed buprenorphine in the ED for approximately one year from the point at which providers began to prescribe buprenorphine (June 15, 2018–June 15, 2019). Patients were not included in the study if they were <18 years old at index visit or if they were pregnant at any time within one year of the index visit. Additionally, we also excluded patients who did not have any other contact with the study site healthcare system within one year of the index visit, as many such patients were determined to be transient. We decided that including such patients in the study could erroneously skew results toward decreased healthcare utilization. Pregnant patients were excluded because it was determined that subsequent ED visits and hospitalizations were likely to skew results as well.

We then reviewed the charts of all patients who formed the buprenorphine group. Data were double-entered onto an abstraction form with standardized coding by medical and undergraduate students who had undergone a general electronic health record (EHR) training session, followed by a study-specific training session provided by author JN.^22^ Abstractors were not blinded to the study hypothesis. We obtained demographic data (age, race, gender), as well as all opioid-related International Classification of Diseases, 10^th^ Revision (ICD-10) codes (including F11.10: opiate abuse, F11.93/F11.23: opiate withdrawal and T40.2: opiate overdose) associated with the patients’ diagnoses during their index visit. No pieces of data were found to be missing once double entry was complete, and conflicting data were addressed by review by senior authors. Abstractors’ progress was assessed at one-month intervals, and accuracy was assessed by comparing data reported by paired abstractors. No formal inter-interpreter reliability analysis was performed.

Next, we specifically searched the EHR for all patients who presented to the site ED during the study period and were diagnosed based on at least one of the opioid-related ICD-10 codes found in the buprenorphine group during their visit. We screened this larger cohort of patients to ensure that they had not been prescribed buprenorphine. Potential controls were then sorted by demographic variables, and the most closely matched control was selected for each member of the buprenorphine group on the basis of gender, age and race. We attempted to select controls with the exact age and gender of buprenorphine patients, and to match by race whenever possible, although the study site’s patient population was largely racially homogeneous and White.

Outcome measures obtained for buprenorphine and control patients included the following: hospitalization rate in the 12 months following index visit; ED visit rate in the 12 months following index visit; and opioid overdose rate in the 12 months following index visit. Additionally, we classified each hospitalization and ED visit as either opioid- or non-opioid related. An opioid-related hospitalization or ED visit was defined as either being the result of opioid use (ie, overdose, withdrawal) or a direct sequela of opioid use (ie, injection-site cellulitis, endocarditis). Classification disagreements between reviewers were adjudicated by the senior author (JN).

Data were de-identified before analysis. We assessed intergroup differences in demographic variables using two-sided t-test and chi-square test, using an alpha of 0.05 to denote statistical significance. Differences in event occurrences between groups were assessed with Poisson regression. We used Stata version 15.1 (Statacorp, College Station, TX) for analysis. Given that the sample size was fixed because it was a convenience sample, formal power analysis was not performed.

## RESULTS

A total of 83 patients were prescribed buprenorphine within the study time frame. Of those patients 24 were excluded due to transience or pregnancy. Ultimately 59 patients were included to form the buprenorphine group, with 58 matched controls (one match served for two of the buprenorphine group due to a lack of eligible subjects with similar demographics) for an overall total of 117 subjects. The groups were well-matched on age, race and gender, and did not differ significantly in any of these variables. See Table 1 for full demographic data.

Patients in the buprenorphine group experienced a total of 137 ED visits, with a median one visit per patient (range 0–8). The group experienced 29 total hospitalizations, with a median 0 hospitalizations per patient (range 0–4). The group experienced 17 total opioid overdoses, with a median 0 overdoses per patient (range 0–3). Patients in the control group experienced a total of 222 ED visits, with a median two ED visits per patient (range 0–33). The group experienced a total of 84 hospitalizations, with a median 0.5 hospitalizations per patient (range 0–8). The group experienced 16 total overdoses, with a median 0 overdoses per patient (range 0–3).

The buprenorphine group experienced a significantly lower 12-month ED visit rate (IRR = 0.61; 95% CI, 0.49, 0.75). The buprenorphine group also experienced a significantly lower 12-month hospitalization rate compared to the control group (IRR = 0.34; 95% CI, 0.22, 0.52). No significant difference between the groups was found for overdoses (IRR = 1.04; 95% CI, 0.53, 2.07)). See Table 2.

When ED visits and hospitalizations were stratified to either opioid- or non-opioid-related, differences between the groups persisted. Patients in the buprenorphine group experienced lower rates of both opioid-related and non-opioid-related hospitalizations (IRR = 0.34; 95% CI, 0.22, 0.52) and IRR = 0.08; (95% CI, 0.02, 0.35, respectively). Buprenorphine group patients also experienced significantly lower rates of non-opioid-related ED visits (IRR = 0.46; 95% CI, 0.32, 0.66)], but did not experience lower rates of opioid-related ED visits (IRR =1.10; 95% CI, 0.77, 0.58)].

## DISCUSSION

This is the first retrospective, matched cohort study to examine whether buprenorphine prescription by an emergency physician in a community ED decreased healthcare utilization in OUD patients. Our results suggest that training emergency care providers to prescribe buprenorphine decreases patient healthcare utilization and does not increase opioid overdose rates compared to controls. Subjects in the buprenorphine group experienced significantly lower rates of ED visits and hospitalizations in the 12 months following buprenorphine prescription by an emergency care provider. Additionally, the IRR for overdoses between the two groups was nearly 1 (1.04), suggesting that buprenorphine prescription by emergency care providers did not increase overdose rates.

Much has been written recently regarding buprenorphine prescription by emergency care providers, reflecting its potential as a gateway to MAT for OUD patients. Multiple studies, such as those by Kaucher et al., Edwards et al., and Dunkley et al. found that buprenorphine induction by emergency physicians was effective in encouraging 30-day follow-up in MAT clinics, although success rates varied (49% [Kaucher] vs 63% [Edwards]).^16^, ^18^–^19^ Furthermore, D’Onofrio et al.’s randomized clinic trial found that, compared to brief intervention and referral to treatment, ED buprenorphine induction resulted in significantly higher 30-day MAT enrollment rates, as well as decreased self-reported opioid use and utilization of inpatient addiction treatment.^21^

Both Lowenstein et al. and Fox et al. described potential barriers to implementation of MAT prescription by emergency physicians.^23^, ^24^ Lowenstein et al. surveyed emergency physicians in two urban, academic EDs regarding physician preparedness to prescribe buprenorphine and perceived barriers to its administration. They found that some reported barriers, such as patient social barriers and lack of patient interest in treatment, were consistently reported by all providers. Reporting of other barriers, such as comfort initiating buprenorphine and perceived safety of buprenorphine, was significantly higher in physicians who had not undergone X-waiver training.^23^ Fox et al. reviewed the current status of ED buprenorphine prescription in the US as well as barriers to ED-initiated buprenorphine therapy. They found that healthcare provider stigma toward patients who use drugs presents a major barrier to MAT prescription, as well as misconceptions regarding X-waiver training.^24^

Our experience is in line with these findings. Anecdotally, our emergency care providers were unsure of their knowledge regarding opioid MAT before X-waiver training but felt more comfortable discussing MAT with patients and prescribing buprenorphine after training. Additionally, the experience of receiving X-waiver training and prescribing MAT motivated some providers to begin working in MAT clinics.

Our study is unique in that it is one of the few to track healthcare utilization after buprenorphine prescription. Hu et al. tracked six-month ED visits and hospitalizations and found that study patients who remained enrolled in MAT experienced significantly decreased rates of six-month ED visits compared to patients who dropped out.^20^ Additionally, our study is one of the few to take place in a rural, community setting, and our case-matched control design allowed for effective intergroup comparison of healthcare utilization.

## LIMITATIONS

This study had several limitations, most importantly its retrospective methodology, which prevents the assumption of causality and limits our conclusions to hypothesis generating. It is also limited by its small sample size, although our primary findings achieved both statistical significance and clinical relevance. Furthermore, abstractors were not blinded to the study hypothesis, and no formal inter-abstractor reliability analysis was performed, although data was entered by two abstractors for each patient, and any discrepancies were adjudicated by a senior author. We were unable to track subjects’ progress in MAT as subjects referred to multiple MAT clinics, some of which were not affiliated with the study site. Neither were we able to obtain mortality data for the patient cohort for this unfunded study, given that our community site does not maintain a contract with the Social Security Administration Death Master File. While no patients in the cohort presented to the ED in arrest, or had a death noted in EHR queries, there is certainly a possibility of patients dying outside a healthcare facility without being brought to the ED or dying in a different healthcare system.

Although both groups were similar in terms of demographics and ICD-10 diagnoses at index visit, no formal protocol was in place to screen patients for buprenorphine treatment. Therefore, it is possible that the buprenorphine and control groups differed in motivation levels, with some proportion of the buprenorphine group actively seeking help and effectively self-selecting. Our study is also limited by the possibility that patients experienced events or hospitalizations at outside healthcare systems, although the study site’s position as the major healthcare system in its six-county area is a potentially ameliorating factor. Lastly, patients in the control group were selected on the basis of opioid-related ICD-10 codes found in the buprenorphine group. Although the buprenorphine group was found to have been diagnosed with a variety of opioid-related ICD-10 codes (ranging from opioid abuse, to overdose, to withdrawal), it is possible that this mechanism introduced some measure of bias.

## CONCLUSION

In this retrospective study, we found that opioid use disorder patients prescribed buprenorphine in a rural, community ED had lower 12-month ED visit and hospitalization rates compared to matched controls, but no change in overdose rate. As the opioid crisis shows few signs of declining, our findings reinforce the potential of ED buprenorphine prescription as a means of combating the crisis. Further research is needed to ensure the safety and examine the long-term efficacy of this technique.

## Figures and Tables

**Table 1:** Demographics of the study cohort.

	Total cohort (n = 117)	Buprenorphine (n = 59)	Control (n = 58)
Age (Mean [SD])	33.4 (8)	33.4 (8)	33.4 (8)
White (95% CI)	109 (93.2%, 88.6%, 97.7%)	53 (89.8%, 82.1%, 97.5%)	56 (96.6%, 91.9%, 100%)
Male (95% CI)	72 (61.5%, 52.6%, 70.3%)	37 (62.7%, 50.4%, 75.0%)	35 (60.3%, 47.8%, 72.8%)

*SD*, standard deviation; *CI*, confidence interval.

**Table 2:** Average healthcare utilization for experimental and control groups.

	Buprenorphine	Control	IRR
1-year Hospitalizations (total, median [range])	29, 0 (0–4)	84, 2 (0–33)	0.34 (95%CI 0.22, 0.52)
1-year ED visits (total, median [range])	139, 1 (0–8)	222, .5 (0–8)	0.61 (95%CI 0.49, 0.75)
1-year Overdoses (total, median [range])	17, 0 (0–3)	16, 0 (0–3)	1.04 (95%CI 0.53, 2.07)

*IRR*, incidence rate ratio; *ED*, emergency department.
